# Development and initial clinical testing of a multiplexed circulating tumor cell assay in patients with clear cell renal cell carcinoma

**DOI:** 10.1002/1878-0261.12931

**Published:** 2021-03-03

**Authors:** Rory M. Bade, Jennifer L. Schehr, Hamid Emamekhoo, Benjamin K. Gibbs, Tamara S. Rodems, Matthew C. Mannino, Joshua A. Desotelle, Erika Heninger, Charlotte N. Stahlfeld, Jamie M. Sperger, Anupama Singh, Serena K. Wolfe, David J. Niles, Waddah Arafat, John A. Steinharter, E. Jason Abel, David J. Beebe, Xiao X. Wei, Rana R. McKay, Toni K. Choueri, Joshua M. Lang

**Affiliations:** ^1^ Carbone Cancer Center University of Wisconsin‐Madison WI USA; ^2^ Department of Medicine University of Wisconsin‐Madison WI USA; ^3^ Department of Biomedical Engineering University of Wisconsin‐Madison WI USA; ^4^ Lank Center for Genitourinary Oncology Dana‐Farber Cancer Institute Harvard University Boston MA USA; ^5^ Moores Cancer Center University of California San Diego La Jolla CA USA

**Keywords:** biomarkers, circulating tumor cells, clear cell renal cell carcinoma, exclusion‐based sample preparation, pharmacodynamic, prognostic

## Abstract

Although therapeutic options for patients with advanced renal cell carcinoma (RCC) have increased in the past decade, no biomarkers are yet available for patient stratification or evaluation of therapy resistance. Given the dynamic and heterogeneous nature of clear cell RCC (ccRCC), tumor biopsies provide limited clinical utility, but liquid biopsies could overcome these limitations. Prior liquid biopsy approaches have lacked clinically relevant detection rates for patients with ccRCC. This study employed ccRCC‐specific markers, CAIX and CAXII, to identify circulating tumor cells (CTC) from patients with metastatic ccRCC. Distinct subtypes of ccRCC CTCs were evaluated for PD‐L1 and HLA‐I expression and correlated with patient response to therapy. CTC enumeration and expression of PD‐L1 and HLA‐I correlated with disease progression and treatment response, respectively. Longitudinal evaluation of a subset of patients demonstrated potential for CTC enumeration to serve as a pharmacodynamic biomarker. Further evaluation of phenotypic heterogeneity among CTCs is needed to better understand the clinical utility of this new biomarker.

## Graphical abstract

Circulating tumor cells (CTCs) were evaluated for their expression of programmed death ligand‐1 (PD‐L1) and human leukocyte antigen class I (HLA‐I), so that biomarkers of therapeutic resistance of clear cell renal cell carcinoma can be developed. CTCs were captured with antibody‐conjugated magnetic beads against EpCAM and CAIX then probed for CAXII and CK expression using microfluidic technology.

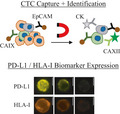

**Abbreviations**
AUC: area under the curve
CA: carbonic anhydrase
ccRCC: clear cell RCC
CK: cytokeratin
CTC: circulating tumor cell
DP: douple‐positive
EpCAM: epithelial cell adhesion molecule
ESP: exclusion‐based sample preparation
FBS: fetal bovine serum
HLA‐I: human leukocyte antigen class I
ICIs: immune checkpoint inhibitors
Ipi: ipilimumab
LR: likelihood ratio
Nivo: nivolumab
PBMCs: peripheral blood mononuclear cells
PBS: phosphate buffered saline
PD‐L1: programmed death ligand‐1
PMPs: paramagnetic particles
RCC: renal cell carcinoma
RECIST: response evaluation criteria in solid tumors
ROC: receiver operating characteristic
S+: single‐positive
TKIs: tyrosine kinase inhibitors
VEGF‐R: vascular endothelial growth factor receptor
VERSA: versatile exclusion‐based rare sample analysis
WBC: white blood cell

## 1 Introduction

Renal cell carcinoma (RCC) is one of the top ten causes of cancer death in the United States [1]. Approximately 25% of patients present with metastatic disease, and an additional 20‐30% of patients with localized cancer will develop metastatic disease despite curative intent radical nephrectomy [2]. The most common histologic subtype is clear cell RCC (ccRCC), representing approximately 85% of patients diagnosed with RCC [3]. Currently, the standard of care treatment approaches for most ccRCC patients include either a dual regimen of immune checkpoint inhibitors (ICIs) (ipilimumab/nivolumab) or an ICI combined with tyrosine kinase inhibitors (TKIs) targeting the Vascular Endothelial Growth Factor Receptor (VEGF‐R) (e.g., Pembrolizumab/Axitinib) [4]. Unfortunately, there are no predictive biomarkers in ccRCC to guide treatment selection of these different therapeutic options nor to identify early signs of treatment resistance.

Programmed death ligand (PD‐L1) expression in a tumor biopsy from a single lesion has shown predictive value for ICIs in some solid tumors such as lung cancer, but not ccRCC [5, 6, 7, 8, 9]. This may be due to the high levels of spatial tumoral heterogeneity across the primary tumor and metastatic lesions that would not be captured by analysis of a single tumor biopsy [5, 6, 7, 10]. Furthermore, effective PD‐L1 blockade relies on functional antigen presentation on the tumor cell surface, where ~ 40% of patients with ccRCC have downregulated expression of the required human leukocyte antigen class I (HLA‐I) [9, 11]. HLA‐I expression levels have been linked to overall prognosis in ccRCC, as well as specific response rates to TKIs, providing a rationale for evaluating both PD‐L1 and HLA‐I in correlation with response to VEGF‐R TKIs and ICIs [8, 12]. Evaluating these biomarkers on circulating tumor cells (CTCs) could better account for intra‐ and intertumoral heterogeneity, leading to the development of biomarkers with predictive utility for patients with ccRCC.

Previous approaches to evaluate CTCs in ccRCC have shown limited clinical value due to either low detection rate or high false positives. Methods using standard antibodies for CTC capture and identification (i.e., epithelial cell adhesion molecule (EpCAM) and cytokeratin (CK)), which can be downregulated in ccRCC, only identified CTCs in 16‐25% of patients and with an average of 1 CTC per 7.5mL of blood [13, 14, 15, 16, 17, 18]. Other attempts to use nonstandard capture antibodies have encountered lower specificity due to non‐neoplastic cells in the blood (e.g., neutrophil and endothelial cells) being falsely identified as CTCs [19, 20, 21, 22, 23, 24, 25, 26].

We hypothesize that the poor detection rate of CTCs in ccRCC is due to differential expression of the classic markers used for CTC identification (i.e., EpCAM and CK) in traditional CTC assays. Prior studies have found that carbonic anhydrase (CA) genes, such as CAIX and CAXII, are expressed in nearly all ccRCC biopsies, potentially due to hypoxia and VEGF dependence in this disease [3, 27]. CAIX is a known diagnostic marker used for clinical pathologic analysis to diagnose renal cell carcinoma [28, 29, 30], and others have shown elevated CAXII expression in renal cells [31, 32, 33, 34]. Evaluation of these renal‐specific markers identified expression in ccRCC CTCs using multicolor flow cytometry. A microfluidic technology known as exclusion‐based sample preparation (ESP) demonstrating high capture efficiency of low‐affinity targets was utilized to capture ccRCC CTCs with both CAIX and EpCAM [35]. High specificity was achieved by adopting a rigorous white blood cell (WBC) and endothelial cell exclusion panel, and with positive CTC identification that differentiated between CTCs expressing CAXII as opposed to CK [20]. CTC number and PD‐L1/HLA‐I expression was associated with disease response determined by radiographic imaging. CTC heterogeneity was identified in a subset of patients, highlighting the importance of evaluating phenotypic signatures in liquid biopsies for ccRCC. The results from this initial cohort support the integration of these biomarkers in prospective clinical trials.

## 2 Materials and methods

### 2.1 Cell culture

RCC cell lines were maintained at 37 °C and 5% CO_2_. SK‐RC52 (RRID:CVCL_6198) and 786‐O (RRID:CVCL_1051) cell lines were cultured in Corning Cellgro RPMI 1640 Medium (Corning, 10‐040‐CV, Corning, NY, USA) with 10% Fetal Bovine Serum (FBS) (Life Technologies, 10437‐028, Carlsbad, CA, USA), 2% penicillin‐streptomycin (Hyclone, SV30010, Logan, UT, USA), 1% sodium pyruvate (Corning, 25‐000‐CI), and 0.1% beta‐mercaptoethanol. CAKI‐1 (RRID:CVCL_0234) and CAKI‐2 (RRID:CVCL_0235) were purchased from ATCC, and cell lines were cultured in McCoy’s 5A Medium (Modified) media (Hyclone, SH3020001) with 10% FBS and 2% penicillin‐streptomycin. All cell lines were authenticated by short tandem repeat profiling in October of 2018 by UW‐Madison TRIP laboratory.

### 2.2 Patient samples

Fifty eight peripheral blood samples from 29 patients with ccRCC were processed under an IRB approved protocol from either Dana‐Farber Cancer Institute (2017‐1567) (seven samples from seven patients) or the University of Wisconsin‐Madison Carbone Cancer Center (2014‐1214) (51 samples from 22 patients) (Table 1). All patients provided written consent prior to enrollment, and study methodologies conformed to the standards set by the Declaration of Helsinki. Detailed patient characteristics are provided in supplemental materials (Table S1), including treating physician interpretation of radiographic assessment of disease response within closest proximity to the blood draw (average 3.6 weeks, range 0–12 weeks). Peripheral blood mononuclear cells (PBMCs) were isolated from whole blood using a Ficoll gradient and fixed for 15 min as described previously [36]. PBMCs were fixed within 48 h from the time of blood collection.

**Table 1:** Patient Characteristics Summary of clinical characteristics for the cohort of patients evaluated in association with these data sets (*n* = 29). Patients 21–26 are not included in summary statistics for ‘Therapy at Time of Blood Draw’; see Figure 4 for further detail. Additional details for each individual are provided in the supplemental materials

	Patients (*n* = 29)
*Age*	61 (44‐79)
*Gender*	
Male	62% (18)
Female	38% (11)
*Histology*	
Clear Cell	100% (29)
Rhabdoid Features	17% (5)
Sarcomatoid Features	7% (2)
*Fuhrman grade*	
1	0% (0)
2	38% (11)
3	21% (6)
4	24% (7)
NA	17% (5)
*Prior nephrectomy*	52% (15)
*Lines of prior therapy*	
0	41% (12)
1	17% (5)
2	17% (5)
3	14% (4)
≥4	10% (3)
*Metastatic sites at time of blood draw*	
Lung	66% (19)
Liver	34% (10)
Lymph Node	31% (9)
Adrenal	31% (9)
Bone	21% (6)
CNS	17% (5)
Other	48% (14)
*Therapy at time of blood draw*	
TKI	62% (18)
Immunotherapy	10% (3)
Combination	3% (1)
None	7% (2)

### 2.3 Flow cytometry

PBMC protein expression profiling was performed by flow cytometry on an LSR Fortessa (BD Biosciences, San Jose, CA, USA) and analyzed with flowjo software v9.9 (Treestar, OR) courtesy of the University of Wisconsin Flow Core facility. Samples were stained for extracellular markers CAIX (R&D systems, AF2188, Minneapolis, MN, USA, conjugated to PE‐Cy7 with abcam kit ab102903), CAXII (FITC, Cedarlane Labs, 10617‐MM07‐F, Burlington, NC, USA), EpCAM (PE, Abcam, ab112068, Cambridge, MA, USA), CD11b (647, BioLegend, 101218, San Diego, CA, USA), CD14 (APC, BioLegend, 301807), CD34 (647, BioLegend, 343508), CD45 (647, BioLegend, 304018), CD235a (APC, BioLegend, 349113), and a Live/Dead fixable viability dye (violet 510, Tonbo Biosciences, 13‐0870‐T100, San Diego, CA, USA) prior to fixation and permeabilization with BD Cytofix/Cytoperm (BD, 554723). Following fixation and permeabilization, cells were stained for pan‐cytokeratin (C‐11, BioLegend, 628602, conjugated to A790 (Life Tech, A20189)). Putative CTC populations were defined by positive expression of either CAIX, CAXII, CK, or EpCAM and negative for the expression of normal cell markers (described as exclusion markers) CD11b, CD14, CD34, CD45, or CD235a (Fig. S1). Exclusion markers identify myeloid cells (CD11b and CD14), immature hematopoietic and endothelial cells (CD34), mature hematopoietic (CD45), and immature red blood cells and plasma B cells (CD235a). Euler diagrams were used to convey the complex relationships between the different CTC biomarkers: where circles of different sizes represent the sizes of each subpopulation, and the degree of overlap between different circles represents the frequency of cells expressing multiple biomarkers.

### 2.4 CTC capture

ESP technology was used to isolate CTCs from patient samples. ESP enables multiple purification processes in sequence (e.g., cell capture, extracellular staining) with high recovery and low cell loss [35]. Analytes were bound to antibody‐functionalized paramagnetic particles (PMPs) and magnetically drawn across phase boundaries (aqueous/oil interface) using an external magnetic force to isolate the PMP‐bound analyte from the sample. Antibody concentration was tested on live and fixed RCC cell lines to optimize capture efficiency using the ExtractMAX, an automated ESP platform [37].

Patient CTCs were extracted using the VERSA (Versatile Exclusion‐based Rare Sample Analysis) platform, a manual ESP technology [36]. This ESP technology captures low‐affinity analytes with greater efficiency than tube‐based methods, but to further maximize yield, cells were bound to antibodies to CAIX (R&D Systems, BAF2188) and EpCAM (R&D Systems, AF960) before being bound to large PMPs, SeraMag Speedbeads (GE Life Sciences, 21152104010150, Boston, MA, USA), to minimize steric hindrance [38]. CTCs were captured with both CAIX and EpCAM to ensure retention of cells traditionally considered CTCs, but to also target the large frequency of cells observed to express CAIX in the flow cytometry experiments (see Fig. S2 for cell line confirmation of the functionality of this dual antibody approach).

### 2.5 CTC staining and fluorescence microscopy

Captured cells were first stained with Hoechst, antibodies against CAXII (Cedarlane Labs, 10617‐MM07‐F), CD34 (Biolegend, 343505), CD45 (Biolegend, 304008), CD66b (Biolegend, 305106), and either PD‐L1 (Abcam, ab205921) or HLA‐I (Biolegend, 311416). A donkey anti‐rabbit IgG (Biolegend 406414) secondary stain was used with the PD‐L1 antibody prior to intracellular staining. Following extracellular staining, cells were permeabilized with BD Perm/Wash buffer (BD Biosciences, 51‐2091KZ) and stained with an Alexa Fluor 790 conjugated pan‐CK (C‐11, Biolegend, 628602).

The CTCs were identified after isolation using fluorescent antibodies against both CK and CAXII, which were maintained on separate fluorescent channels to evaluate the heterogeneity of different CTC subpopulations (see Table S3 for an overview of which antibodies were used at which steps in the methods). The combination of both CAIX at the capture step, with CAXII at the identification step maximizes the specificity of CTC identification using the alternative markers, ensuring the evaluation of a population with a truly renal cancer origin.

After staining, the samples were washed three times with phosphate buffered saline (PBS), and then imaged with a 10X objective using a Nikon Eclipse Ti‐e fluorescent microscope (Nikon, Minato City, Japan) with NIS‐Elements AR 4.51.01 software (Nikon). Samples were transferred to a glass slide for high resolution imaging using a 40X apo objective. Imaging of isolated CTCs occurred within 24 h of fixation.

### 2.6 Image analysis

Patient CTC images were analyzed using NIS‐Elements AR Analysis 4.51.01 (Nikon). Using an automated sequence of image analysis algorithms, background fluorescence was rolling ball subtracted prior to setting a binary threshold defining a cell’s boundary. Thresholds were set for positive expression of CAXII, cytokeratin, exclusion (CD34, CD45, and CD66b), PD‐L1, and HLA‐I using the principles of clustering as in flow cytometry [20, 39]. CTCs were defined as a cell with an intact nucleus, exclusion negative, and CAXII or CK positive. Captured cells that were negative for exclusion markers and also negative for CAXII and CK were not counted as CTCs. Each putative CTC underwent rapid manual review to confirm the absence of potentially interfering image artifacts. Analysts were blinded to patient clinical data until all CTC analysis was finalized.

PD‐L1 and HLA‐I expression was assessed on CTCs to evaluate the feasibility and sensitivity of the assay to detect differences in expression among patients. Each individual CTC was quantified for the expression levels of both PD‐L1 and HLA‐I after subtracting the average background fluorescence to account for variability in washing step efficiency. These single‐cell results were then analyzed to generate summary statistics conveying both the percentage of cells with positive expression (% positive) as well as the average expression of each biomarker in each CTC subpopulation identified from each patient sample. Positive expression was defined as expression above a set cutoff based on population clustering, similar to flow cytometry [20, 39].

### 2.7 Statistical analysis

Data were compiled in graphpad prism 7.01 (San Diego, CA, USA) for statistical comparisons. One‐way ANOVAs with multiple comparisons were performed across the different conditions for the cell line capture optimization. A two‐tailed *t*‐test assuming unequal variance was performed on total enumeration between patients who were progressing on their current therapy and patients who were stable or responding on their current therapy, with *P* < 0.05 considered statistically significant. Receiver operating characteristic (ROC) curves were performed in graphpad prism, setting responding patient samples as the control group and progressing patient samples as the patient group and evaluating the relationship with a confidence level of 95%. Patient samples were categorized as responding or progressing based on the treating physician’s interpretation of radiographic assessment. Optimal diagnostic cutoffs were determined based on the cutoff associated with the highest likelihood ratio value. A ROC curve with a *P*‐value of < 0.05 was considered a statistically significant association with disease response or progression compared to the reference curve of a completely random classifier. Area under the curve (AUC) was calculated from ROC curves within Prism.

## 3 Results

### 3.1 Heterogeneity of ccRCC markers on cells in circulation

**Fig. 1:**
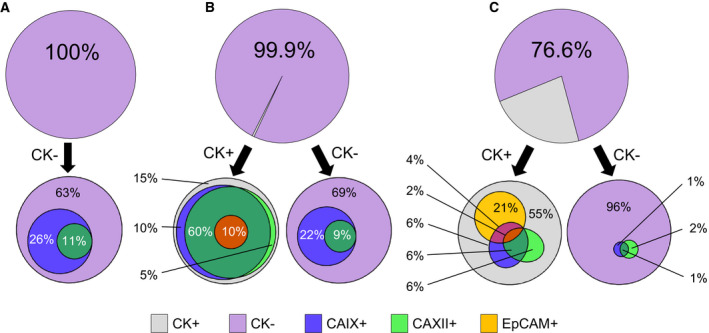
Heterogeneity of RCC biomarkers on cells in circulation. Flow cytometric evaluation of the frequency of expression of different biomarkers on CTCs from n = 3 different patients (A, B, or C). The first row of pie graphs represents the distribution of CK‐positive CTCs (gray) vs. CK‐negative / exclusion channel negative cells (purple). The second row of Euler diagrams portrays the frequency of expression of other markers of renal cancer origin (CAIX, CAXII, and EpCAM) within the CK‐positive vs. CK‐negative / exclusion negative cell fractions. Overlapping circles indicate the co‐expression of different biomarkers on the same cells. Subpopulation frequencies are rounded to the nearest whole percent, with percentages < 0.5% excluded. Exact percentages are provided in Supplementary Table 2.

Potential markers for CTC identification in ccRCC were identified by evaluating blood samples collected from six patients with metastatic ccRCC using multicolor flow cytometry (Table S2). Cellular profiling of samples from three representative patients (Fig. 1) demonstrates the extensive heterogeneity of CTC markers: where a large frequency of exclusion channel negative cells (CD11b−, CD14−, CD34−, CD45−, CD235a−) did not simultaneously express all tumor‐specific markers (CK, EpCAM, CAIX, and CAXII). CTC subpopulations varied in the degree of co‐expression of tumor‐specific markers between different patients as shown by Euler diagrams. Cells positive for exclusion markers represent potential confounding populations and were excluded from this phenotypic analysis. CK+ cells were only detected in 4 out of 6 patients (average frequency of 6% of the exclusion cells; range 0.1‐23.4%) and CK− cells were detected in all 6 patients (average frequency of 96%; range 76.6‐100.0) (Table S4). CK+/EpCAM+ cells were detected in 3 patients (average rate of 14%; range 6.3–27.1%), whereas CK−/EpCAM+ cells were detected in 4 patients (average rate of 1%; range 0.2–2.3%).

CAIX and CAXII were detected in samples from all 6 patients within both CK+ and CK− cells. In addition to being expressed by CTCs from all samples, CAIX and CAXII were expressed on a higher percentage of cells on average compared to EpCAM. CAIX was expressed with an average frequency of 75% within CK+ and 38% within CK− cells. CAXII was expressed with an average frequency of 72% within CK+ and 7% within CK− cells. There was one CK+/EpCAM+ cell population in a single patient that did not co‐express CAIX and CAXII, which suggests that CK and EpCAM retain some limited utility to identify CTC subtypes in a diverse patient population.

### 3.2 ccRCC CTC enumeration correlates with clinical progression

**Fig. 2:**
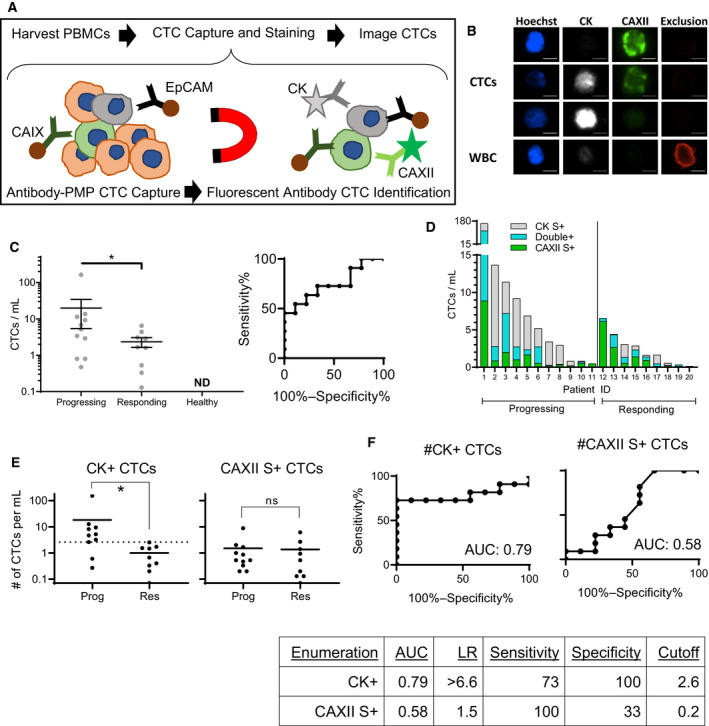
Representative Images and Clinical Correlations of CTC Enumeration. (A) Schematic overview of the method used to isolate and identify CTCs from whole blood. (B) Three representative CTCs and one WBC from a patient blood sample. Examples of CTCs with intact nuclei that were CAXII S+ (top), Double+ (middle), CK S+ (bottom), all Exclusion. Scale bars represent 5 microns. (C) Enumeration of all CTCs for *n* = 20 patients and *n* = 2 healthy donors where each symbol represents the number of CTCs from one patient. Significance was determined by a one tailed Mann‐Whitney test p < 0.05 (*). Error bars represent standard error. ROC curve showing sensitivity and specificity of total CTC number to differentiate between patients whose disease was progressing vs responding. (D) CTC enumeration separated by phenotype for the same n = 20 patients: CAXII single+ (green), double+ (blue), and CK single+ (gray). Percentages of different CTC subpopulations are listed in supplementary table 2. (E) two‐tailed Mann‐Whitney tests and (F) ROC evaluation of the ability of the different populations of CTCs to differentiate between therapeutic progression or response for the same *n* = 20 patients. ROC curve parameters are tabulated in table below ROC curves.

The flow cytometry experiments demonstrated that the CA antigens were both co‐expressed and independently expressed by different populations of cells. However, flow cytometry does not enable manual review of cell staining which precludes the ability to remove any potentially interfering image artifacts, which is particularly important to improve specificity of rare cell analytics. Both EpCAM and CAIX were used to improve the sensitivity of CTC capture using ESP technology, then combined with fluorescence microscopy for CK and CAXII protein staining (Fig. 2A). Solid tumor biopsy tissue from 8 of the patients in this study (Patient ID 9, 13, 16, 18, 22, 24, 25, and 26) were stained for CAIX, and all 8 samples showed positive expression of CAIX, rationalizing this approach to capturing CTCs with the CAIX antibody. CTCs were detected in 100% of samples evaluated from patients with metastatic ccRCC but not in the two samples from healthy donors (Fig. 2C). Patient samples were categorized as progressing or responding based on the treating physician’s interpretation of radiographic scans (Table 1). Samples drawn near the time of radiographic progression had an average of 19.8 CTCs·mL^−1^ (range 0.5–163.1), which was significantly higher than in samples from patients responding to treatment who had an average of 2.3 CTCs·mL^−1^ (range 0.1–6.5, *P* < 0.05). This data set was further evaluated with a ROC curve to identify the cutoff that maximizes specificity without sacrificing sensitivity (Fig. 2C). The optimal cutoff for differentiating between progressing and responding samples was 4.8 total CTCs·mL^−1^, which provided a sensitivity of 55% and a specificity of 89% with a likelihood ratio of 4.9 and a *P*‐value less than 0.05 suggesting a statistically significant association with disease progression.

We hypothesized that the composition of different CTC subpopulations may reflect intralesional tumor heterogeneity and evaluated their independent association with clinical outcomes. Specifically, CAXII+ CTCs lacking expression of cytokeratins may identify a less differentiated and more aggressive phenotype, and as such, CTCs were divided into those with CAXII expression only [CAXII single positives (CAXII S+)] vs. those expressing CK [either CK single‐positive (CK S+) or CK/CAXII double‐positive (DP)]. Heterogeneity in the frequency of CK and CAXII subpopulations was observed between different patients (Fig. 2D). ROC curve evaluations of two groups of CTCs (CAXII S+ vs. CK+ (either CK S+ or DP)) revealed that the number of CK+ CTCs (2.6/mL optimal cutoff) significantly correlated with radiographic progression (P < 0.05), whereas the number of CAXII S+ CTCs did not (*P* > 0.05) (Fig. 2E and F), demonstrating the value of considering CTC heterogeneity in ccRCC CTC diagnostics.

### 3.3 Evaluation of immune evasion markers on CTCs in ccRCC

**Fig. 3:**
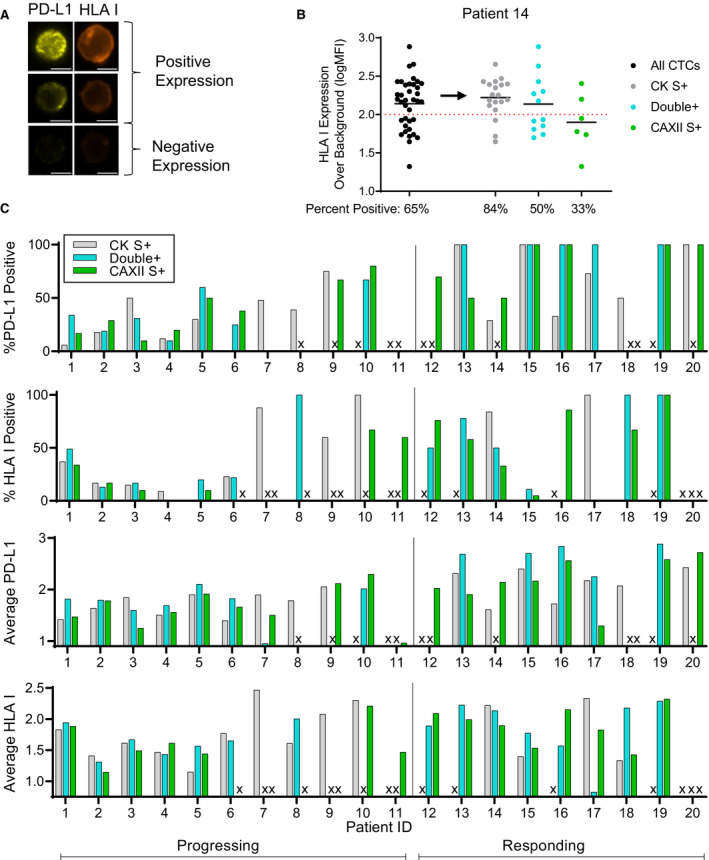
Quantification of Immunotherapy Biomarkers on CTCs. (A) Positive and negative expression of PD‐L1 and HLA‐I expression on CTCs is shown with scale bars representing 5 microns. (B) Biomarker evaluation was performed as a single ‘All CTCs’ (black) population as well as by each subpopulation, CK single+ (gray), double+ (blue), and CAXII single+ (green), where each dot represents a single CTC. Average CTC expression is represented by a black bar through each population, and % positive is defined as the frequency of CTCs with biomarker expression above the red dotted line cutoff for positivity. Representative dataset shown from one patient sample. C) The % positive and average expression of CTCs from each patient were determined for PD‐L1 and HLA‐I for cohort = 20 patients. Samples without CTCs are indicated with an ‘X’.

PD‐L1 and HLA‐I expression was assessed on CTCs as potential pharmacodynamic biomarkers of ICIs and TKIs that may also reflect phenotypic heterogeneity in ccRCC. Expression of these biomarkers revealed heterogeneous expression of PD‐L1 and HLA‐I at both the single‐cell level as well as between different CTC subpopulations (Fig. 3A and B). CTCs from a representative sample showed a broad range of expression for HLA‐I, with 65% of the CTCs having positive expression (Fig. 3B). However, evaluating each CTC subpopulation revealed different expression patterns. CK S+ (gray) CTCs had, in general, higher expression of HLA‐I with 84% having positive expression. Double+ (blue) CTCs segregated into two populations, one positive and one negative, that resulted in an average expression that was similar to that of all CTCs, but a %positive value of only 50%. Finally, the CAXII S+ (green) CTCs had substantially lower expression with only 33% of these CTCs having positive expression. This patient (Pt 14) demonstrates how these heterogeneous CTC subpopulations can provide unique information that is lost if not evaluated independently. Heterogeneous expression of PD‐L1 and HLA‐I was observed across the 20‐patient cohort (Fig. 3C), where samples from many patients had heterogeneous expression of these biomarkers between the different CTC subpopulations.

**Fig. 4:**
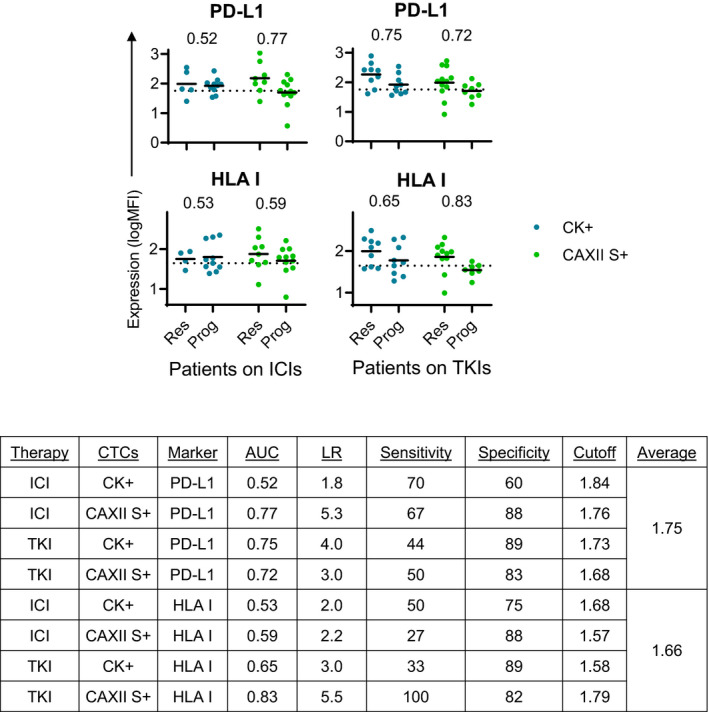
Clinical Utility of Biomarker Evaluation with Different CTC Populations. Scatter plots showing the average expression of either PD‐L1 or HLA‐I on either CK + or CAXII S + CTCs in patients either responding or stable (Res) vs. progressing (Prog) on either ICIs or TKIs. AUC values annotated on graphs are a measure of the ability of the assay to identify patients who are progressing. Data represent 42 total samples from 24 unique patients.

Consistent with the hypothesis that CAXII+ CTCs lacking expression of CK may identify a less differentiated and more aggressive phenotype, the expression of PD‐L1 and HLA‐I in CTC subpopulations differentially correlated with treatment response. Samples were categorized by disease response, either responding or progressing, and on either ICI or VEGF‐R TKI, then ROC curve evaluated for the average CTC expression of either PD‐L1 or HLA‐I (Fig. 4). From 52 blood samples evaluated from a total of 26 patients, 20 samples were collected from patients on ICIs (8 responding; 12 progressing); 22 samples were collected from patients on VEGF‐R TKIs (13 responding; 9 progressing); and 10 samples were excluded from this analysis because they were not collected from patients who had been on any therapy for at least 3 weeks. Of the samples that were collected from patients during treatment, response to ICI therapy was only significantly correlated with expression of PD‐L1 on CAXII S+ CTCs and not on CK+ CTCs (AUCs of 0.77 vs. 0.52). Patient response to VEGF‐R TKIs correlated only with HLA‐I on CAXII S+ CTCs and not on CK+ CTCs (AUCs of 0.83 vs. 0.65). These data represent a small initial cohort of patients (42 total samples from 24 unique patients) but highlights the potential differences in clinical relevance of the different subpopulations of CTCs.

### 3.4 Longitudinal evaluation of CTC subpopulations to identify potential pharmacodynamic biomarkers

**Fig. 5:**
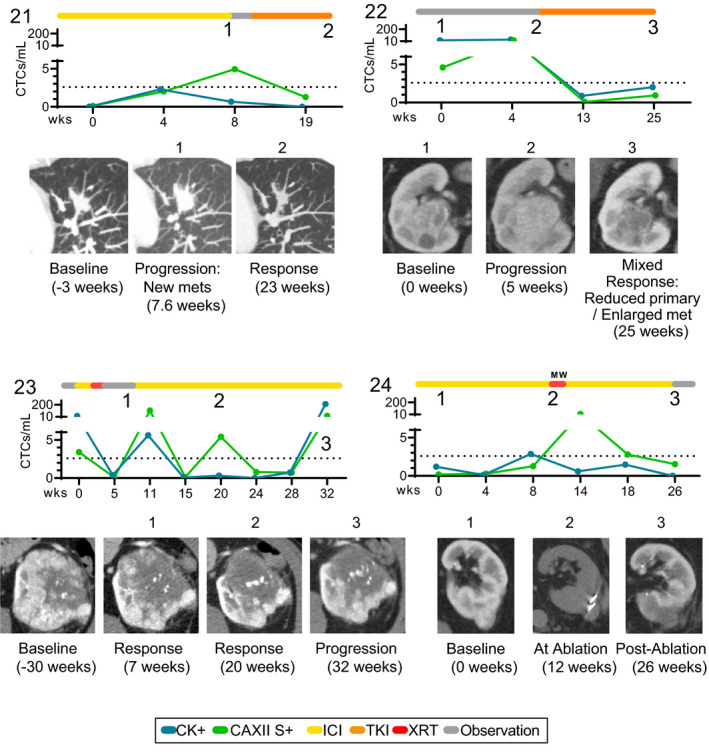
Longitudinal Sampling of CTCs. CTC biomarker evaluation of 4 patients (#21‐24) over time were compared to therapeutic history (colored bars) and radiographic assessment of response (numbers on graphs and corresponding radiographic scan images) for each patient. Graphs represent enumeration for each CTC population. Dashed line represents the optimal cutoff for CK + CTC number (2.6) identified in Figure 2.

The accessibility of CTCs through a blood draw presents new opportunities to develop and evaluate pharmacodynamic biomarkers of disease response as well as emerging signs of resistance. Four patients donated multiple blood samples longitudinally during their treatment course for evaluation of CTCs (Fig. 5). Patient 21 was receiving nivolumab for nearly two months prior to their first CTC collection (indicated as week 0 on the graph). CT scans at the week 8 blood draw showed radiographic progression with increased size of a known endovascular metastasis and a new pulmonary metastasis. CAXII S+ (green) and CK+ (blue) CTCs increased after the initial blood draw correlating with radiographic progression at week 8. Nivolumab was subsequently discontinued and the patient was started on cabozantinib. Both CTC subpopulations decreased after starting cabozantinib, correlating again with radiographic response to cabozantinib at week 19, demonstrating enumeration could potentially be used to track disease status and serve as an early indicator of disease progression or response to treatment.

Patient 22 was on cabozantinib but treatment was held due to treatment related side effects prior to the first CTC sample collection (labeled as week 0 in Fig. 5). High numbers of both CTC subpopulations were observed in the first CTC sample correlating with radiographic progression (based on CT scans at week 5). Once the patient resumed cabozantinib, there was a rapid decline in both CTC subpopulations that corresponded with clinical symptom improvement (pain and fatigue). Twenty weeks after restarting therapy with cabozantinib (week 25), radiographic imaging demonstrated evidence of a mixed response with reduction of the primary renal mass, but growth in a pre‐existing peritoneal nodule which corresponded to slight rises in both CTC subpopulations.

Prior to his first CTC sample collection, patient 23 initially presented with primary ccRCC with a single brain metastasis that was surgically resected, with pathologic confirmation of central nervous system metastasis of ccRCC. He recovered from surgery and completed postoperative radiation to the surgical resection bed. He was considered for cytoreductive nephrectomy as his large renal mass was his main tumor. Unfortunately, he had rapid disease progression with an increase in the size of his primary renal mass, new lung metastases, as well as development of multiple new brain metastases. He underwent radiosurgery to his multiple brain metastases and was started on ipilimumab and nivolumab (Ipi/Nivo). His first CTC sample was collected before starting Ipi/Nivo treatment and demonstrated elevated levels of both CTC subpopulations. Only one week after the first infusion of Ipi/Nivo, he developed new onset neurologic symptoms and was found to have new brain metastases and an intramedullary spinal cord drop metastasis. He was treated with whole brain radiation and radiation to the spine, and Ipi/Nivo was held while he received radiation therapy and systemic steroids. Shortly after completion of radiation therapy, Ipi/Nivo was resumed and imaging performed at week 5 demonstrated radiographic response and corresponding reduction in CTC number. At week 11, there was a dramatic expansion of CAXII S+ CTCs (from 0.07 to 106.4 CTCs·mL^−1^), along with a smaller rise in CK+ CTCs (from 0.4 to 5.6 CTCs·mL^−1^), which reached above the 2.6 CK+ CTCs·mL^−1^ cutoff. Further treatment with ICI was associated with radiographic evidence of response with decrease in the size and enhancement of the primary renal mass and subsequent decline in both CTC subpopulations (week 24). There was a flare of CAXII S+ CTCs that occurred at week 20, shortly after transitioning to single agent nivolumab that was not associated with radiographic progression. At week 32, an increase in size and enhancement of the renal mass was noted while he was on single agent nivolumab. This was associated with a significant increase in the total number of both CTC subpopulations.

Patient 24 was treated with nivolumab for 22 months before his first CTC evaluation. At the time of his first CTC collection, his scans showed increase in the size of an interpolar renal mass and a pancreatic lesion (oligo‐progression), leading to microwave ablation of the renal mass at the scan #2 time point (this scan is without contrast and the patient is lying on their side unlike the other two scans). Both CTC subpopulations increased slightly by week 8, also corresponding to slight increase in the size of a different interpolar renal mass (<20% growth, scan images not shown on figure) at week 12. After the microwave ablation, there was a marked increase in CAXII S+ CTCs (from 1.3 to 12.3 CTCs·mL^−1^), but CK+ CTCs decreased, suggesting distinct changes in CTC subpopulations can occur immediately following ablative vs systemic therapies. Scan #3 shows the tumor area is similar in size to the first scan, but the decreased contrast uptake shows the remaining mass is mainly fibrotic scar tissue, indicating the ablation was successful. Longitudinal data from an additional two patients are included in Fig. S3.

## 4 Discussion

Molecular heterogeneity is a hallmark feature of ccRCC, and it has been reported in primary renal masses as well as different metastatic lesions [10]. Single diagnostic biopsies of metastatic foci are not capable of evaluating the intra‐ and intertumoral heterogeneity, which is present in ccRCC [40, 41, 42] and may explain the lack of success in the development of predictive biomarkers for new therapies. CTCs could offer a unique assessment of heterogeneous cancer clones since they are shed from diverse primary and metastatic tumor sites. Using flow cytometry, we identified expression of renal‐specific markers on ccRCC CTCs and utilized a highly sensitive CTC microfluidic platform to confirm the presence of different subsets of CTCs that express different combinations of CK, EpCAM, CAIX, and/or CAXII. These findings likely explain the limited success of prior CTC assays in ccRCC that utilized CK and EpCAM or a limited assessment of normal cells leading to false‐positive results. This assay, to the best of our knowledge, is the first to evaluate CTC phenotypic heterogeneity that may reflect intratumoral heterogeneity in ccRCC.

CK expression in ccRCC tumors has traditionally been considered to have limited diagnostic utility which is reflected in the low frequency of CTCs detected using traditional methods [15, 17, 18, 43]. However, our dataset suggests that CK+ CTCs are frequently detected, and their number correlates with disease progression likely due to the use of a kidney cancer‐specific capture antibody, CAIX. Enumeration of CK+ CTCs significantly correlated with disease progression in this pilot study; however, the number of CTCs that express only CAXII did not. It is unknown if these different subpopulations of CTCs may represent different aspects of tumor biology such as a mesenchymal transition with decreased expression of CK. One hypothesis is that CK+ CTCs may represent clones which are passively shed from growing tumors, but not responding tumors. CAXII S+ CTCs, however, may be less differentiated and more aggressive, and enter the circulation from active intravasation and have greater metastatic potential. Others have shown that CAXII+ cells are more invasive and aggressive in‐vitro [44, 45]. If CAXII S+ CTCs represent a more invasive/aggressive subset, their average PD‐L1 and HLA‐I expression may also be more critical to predicting treatment response. In this report, PD‐L1 expression on CAXII S+ CTC, but not CK+ CTCs, was significantly correlated with disease response to ICIs. Similarly, HLA‐I expression was only significantly correlated with TKI response in CAXII S+ CTCs as opposed to CK+ CTCs. Future studies of the genomic, epigenomic, and transcriptional signatures of these CTC subpopulations will help elucidate biological properties of these subpopulations.

We did not evaluate the degree of co‐expression of PD‐L1 and HLA‐I on the same CTCs, nor the concordance between CTC and solid biopsy expression here. Others have shown that lower expression of HLA‐I on patient biopsy associates with lower response rates to TKIs, which is consistent with our findings [9]. Future studies to evaluate concordance in expression of HLA‐I and PD‐L1 between solid and liquid biopsies may help to clarify the role these molecules may play in the metastatic process as well as potential utility as predictive biomarkers in ccRCC. Assessing PD‐L1 and HLA‐I co‐expression on the same cell could further enhance our biological understanding and diagnostic capabilities. This may be even more important with the recent FDA approval of axitinib/pembrolizumab and the lack of available biomarkers to aid clinicians in deciding between this first‐line combination therapy and ipilimumab/nivolumab. CTC evaluation could provide an added benefit over standard radiographic imaging of accelerating the switch to alternate, potentially more effective therapies. This initial evaluation of PD‐L1 and HLA‐I on different subpopulations of CTCs in ccRCC shows promising correlations with disease response to both ICIs and VEGF‐R TKIs and warrants more in‐depth investigation.

Longitudinal evaluation of CTCs in patients over the course of therapy demonstrates the pharmacodynamic potential of these CTC analytics. Fluctuations in CK+ CTC number seemed to correlate with radiographic evidence of tumor growth, with smaller changes in tumor growth being mirrored with more subtle changes in CK+ CTC number. CAXII S+ CTCs increased in number after radiation, potentially indicating rapid shedding of necrotic tumor cells into circulation that may further reflect the benefit of these interventions. The differential kinetics of these CTC subpopulations further supports the value in evaluating CTC heterogeneity in the development of highly informative diagnostic tools.

Fully understanding the biologic relevance and clinical utility of these different read‐outs of CTC evaluation will require prospective clinical trials. Clinical correlations from this patient population are limited due to the variability in therapeutic regimen, nonstandard assessment of disease status, lack of long‐term follow‐up, and variable intervals between scans and CTC evaluation. Obtaining multiple samples at baseline and prespecified time points throughout the treatment would provide more uniform data to assess CTC changes based on response to treatment. Prospective response evaluation based on imaging (e.g., Response Evaluation Criteria in Solid Tumors (RECIST) criteria) and/or other clinical and laboratory criteria would provide a more standardized disease status assessment to be correlated with CTC findings. Toward this end, this CTC assay is currently being tested in a multisite phase II clinical trial evaluating a response‐based approach to treatment with nivolumab and ipilimumab in patients with advanced ccRCC (NCT03203473) [46]. Further, clinical validation in an independent cohort will be required to confirm the results from the initial prospective patient cohort. Other prospective trials have incorporated these CTC biomarkers including the RadiCaL trial (NCT04071223) of Radium‐223 combined with cabozantinib. Additionally, ongoing efforts are directed at further automation of the method to ensure reproducibility across different laboratories.

## 5 Conclusions

In conclusion, these findings support further evaluation of this novel approach to CTC characterization, which could prove to have prognostic, predictive, and pharmacodynamic utility to advance precision medicine approaches to improve outcomes in patients with advanced ccRCC. We describe the use of novel microfluidic technology, combined with EpCAM and renal‐specific CAIX antibody for high sensitivity CTC capture. High specificity is achieved with a rigorous exclusion antibody panel, and the identification of CTCs with CAXII and CK. Initial clinical results evaluating expression of both PD‐L1 and HLA‐I on ccRCC CTCs found higher expression associated with treatment response. These assays are now being deployed in prospective clinical trials to evaluate as potential predictive or pharmacodynamic biomarkers.

## Conflict of interest

David J. Beebe and Joshua M. Lang hold equity in Salus Discovery LLC, which has licensed technology utilized in the manuscript. D. J. Beebe holds equity in Tasso, Inc., Stacks for the Future, LLC, and Bellbrook Labs, LLC. Rana R. McKay is a consultant/Advisory Board Member for Bristol‐Myers Squibb, Dendreon, Exelixis, Janssen, Pfizer, Novartis, and Tempus; receives institutional research funding from Bayer and Pfizer. Xiao X. Wei receives institutional research funding from Bristol‐Myers Squibb.

## Author contributions

RMB, JLS, HE, BKG, JAD, EH, JMS, and JML conceptualized the ideas; RMB, JLS, EH, JMS, and DJN provided the methods; RMB, JLS, HE, JAD, and EH collected and curated the data, and all authors provided critical feedback and helped review and edit the final manuscript.

## Supporting information

**Table S1.** Patient characteristics.
**Table S2.** Distribution of CTC identification markers (CK, CAIX, CAXII, and EpCAM) amongst exclusion negative cells.
**Table S3.** Overview of which antibodies were used for the different methodologies.
**Table S4.** Composition of CTC sub‐populations for the 20 patient cohort.
**Fig. S1**. Flow cytometry was used to evaluate the expression of different biomarkers on different populations of CTCs.
**Fig. S2.** The percentage of captured cells was evaluated on four RCC cell lines after fixation (A) and live (B) using indirect SeraMag binding with antibodies to CAIX (blue), EpCAM (red), or CAIX and EpCAM (red/blue).
**Fig. S3.** CTC biomarker evaluation of two additional patients (#25 and 26) over time compared to therapeutic history (colored bars) for each patient.

## Data Availability

The data that support the findings of this study are available from the corresponding author jmlang@medicine.wisc.edu upon reasonable request.
