# Supplementary material for: Development and initial clinical testing of a multiplexed circulating tumor cell assay in patients with clear cell renal cell carcinoma
Source: Mol Oncol. 2021 Mar 3;15(9):2330–44. doi: 10.1002/1878-0261.12931 (PMC8410529; doi:10.1002/1878-0261.12931)
Supplement: Supplementary file 1 — Table S1. Patient characteristics. Table S2. Distribution of CTC identification markers (CK, CAIX, CAXII, and EpCAM) amongst exclusion negative cells. Table S3. Overview of which antibodies were used for the different methodologies. Table S4. Composition of CTC sub‐populations for the 20 patient cohort. Fig. S1. Flow cytometry was used to evaluate the expression of different biomarkers on different populations of CTCs. Fig. S2. The percentage of captured cells was evaluated on four RCC cell lines after fixation (A) and live (B) using indirect SeraMag binding with antibodies to CAIX (blue), EpCAM (red), or CAIX and EpCAM (red/blue). Fig. S3. CTC biomarker evaluation of two additional patients (#25 and 26) over time compared to therapeutic history (colored bars) for each patient. [file MOL2-15-2330-s001.pdf]

							Metastatic Sites at Time of Blood Draw								
Patient ID	Age	Gender	Sarcomatoid or Rhabdoid Features	Fuhrman Grade	Prior Nephrectomy	Lines of Prior Therapies	Lung	Liver	Lymph Node	Adrenal	Bone	CNS	Other	Therapy at Time of Blood Draw	Radiographic Assessment in proximity to Blood Draw
1	79	M		2	N	3	X	X					X	None	PD
2	63	M		NA	N	5	X		X		X		X	Lenvatinib	PD
3	60	M		2	Y	0	X						X	Axitinib + Avelumab	PD
4	67	F		3	Y	9	X		X	X				Cabozantinib	PD
5	53	M		2	Y	0							X	Axitinib + Avelumab	PD
6	60	M		4	Y	0	X		X	X	X			Sunitinib	PD
7	54	M		NA	N	3	X		X		X			Cabozantinib	PD
8	79	M		2	Y	1	X							Nivolumab	PD
9	65	M		4	N	2	X							Pazopanib	PD
10	56	M		NA	N	0	X	X	X	X			X	Ipilimumab + Nivolumab	PD
11	79	M		3	N	0			X	X				None	PD
12	59	M		2	Y	0		X	X					Pazopanib	PR
13	63	F		3	Y	1	X	X				X		Pazopanib	PR
14	44	M	Rhabdoid	2	Y	0	X				X			Pazopanib	PR
15	67	F	Sarcomatoid and Rhabdoid	4	Y	1	X	X					X	Nivolumab	PR
16	54	M		NA	N	2		X		X			X	Cabozantinib	PR
17	67	F		NA	N	2					X			Cabozantinib	PR
18	51	F	Rhabdoid	4	N	3	X	X				X		Everolimus + Lenvatinib	PR
19	56	M	Rhabdoid	4	N	3		X		X		X	X	Cabozantinib	PR
20	60	F		2	Y	2		X	X					Cabozantinib	PR
21	51	F		3	Y	1	X							Nivolumab or Cabozantinib*	NA
22	63	F		2	N	0							X	Cabozantinib or Ipilimumab + Nivolumab*	NA
23	52	M	Sarcomatoid	4	N	0	X					X		XRT or Ipilimumab + Nivolumab*	NA
24	64	M		3	Y	0	X			X			X	Nivolumab or XRT*	NA
25	44	F	Rhabdoid	4	N	1	X	X	X	X	X	X	X	XRT or Ipilimumab + Nivolumab*	NA
26	69	F		2	N	0	X						X	Pazopanib	NA
27	53	F		3	Y	4				X				Nivolumab	NA
28	76	M		2	Y	0							X	Sunitinib	NA
29	70	M		2	Y	2	X						X	Axitinib	NA

Supplemental Table 1: Patient characteristics. Patients included in this table represent all patients from all experiments and figures included in this paper. Patients who were either evaluated at multiple blood draw time points (annotated with * after therapy at draw) or who were not included in correlative studies involving radiographic assessment were indicated with NA (not applicable) in the column labeled radiographic assessment at blood draw. PD = Progressive Disease, PR = Partial Response. Fuhrman grade "NA" indicates not available due to biopsy being performed on a metastatic site.

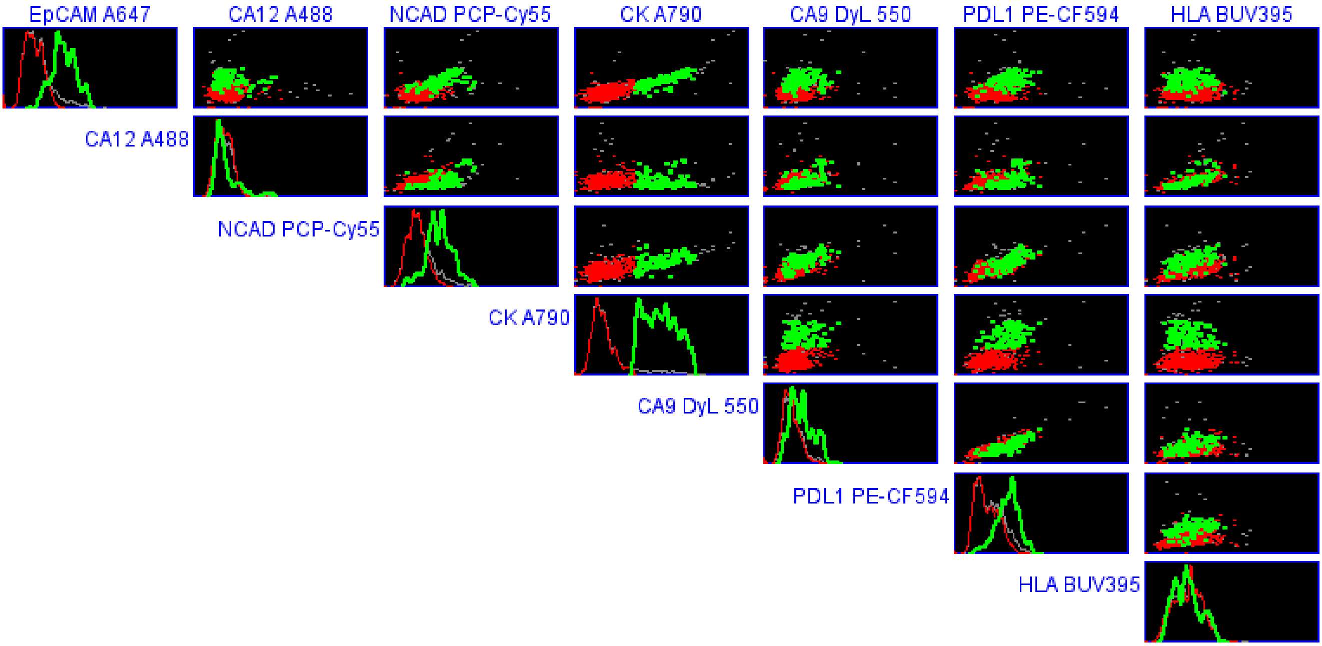

Supplemental Figure 1: Flow Cytometry was used to evaluate the expression of different biomarkers on different populations of CTCs. Putative CTC populations were first identified by the absence of expression of multiple antigens cumulatively describes as “exclusion” markers (CD11b, CD14, CD34, CD45, CD235a). This exclusion negative population was split into groups of either CK positive (green) or CK negative (red) expression, and overlaid onto the exclusion positive population (gray). The grid of scatter plots demonstrates both the distribution of biomarkers within the different CK expressing populations, as well as the mutual vs. differential expression of the CTC identification biomarkers on individual cells.

Patient ID:	7	15	20	27	28	29
**CK+**	0	23.4	0.5	0.1	0	0.4
**CK-**	100	76.6	99.5	99.9	100	99.6
**CK+/EpCAM+**	0	27.1	0	10	0	6.3
**CK-/EpCAM+**	0.3	0.3	0	0.2	0	2.3
**CK+/CAIX+**	0	18.1	100	80	0	100
**CK-/CAIX+**	54.9	2.2	66.2	30.5	36.8	36.3
**CK+/CAXII+**	0	14.1	100	75	0	100
**CK-/CAXII+**	1.1	2.6	6.5	9	10.5	14.7
**CK+/CAIX+/CAXII+**	0	7.5	100	70	0	100
**CK-/CAIX+/CAXII+**	1	0.6	5.1	9.1	10.5	14.1
**CK+/CAIX+/EpCAM+**	0	6	0	10	0	6.3
**CK-/CAIX+/EpCAM+**	0.1	0	0	0.1	0	0.3
**CK+/CAXII+/EpCAM+**	0	1.5	0	10	0	6.3
**CK-/CAXII+/EpCAM+**	0	0	0	0.2	0	0.5
**CK+/CAIX+/CAXII+/EpCAM+**	0	1.5	0	10	0	6.3
**CK-/CAIX+/CAXII+/EpCAM+**	0	0	0	0	0	0.1

Supplemental Table 2: Distribution of CTC identification markers (CK, CAIX, CAXII, and EpCAM) amongst exclusion negative cells. Samples were first separated into CK+ and CK- populations and then evaluated for the remaining biomarkers. Co-expression of markers was investigated to evaluate CTC heterogeneity.

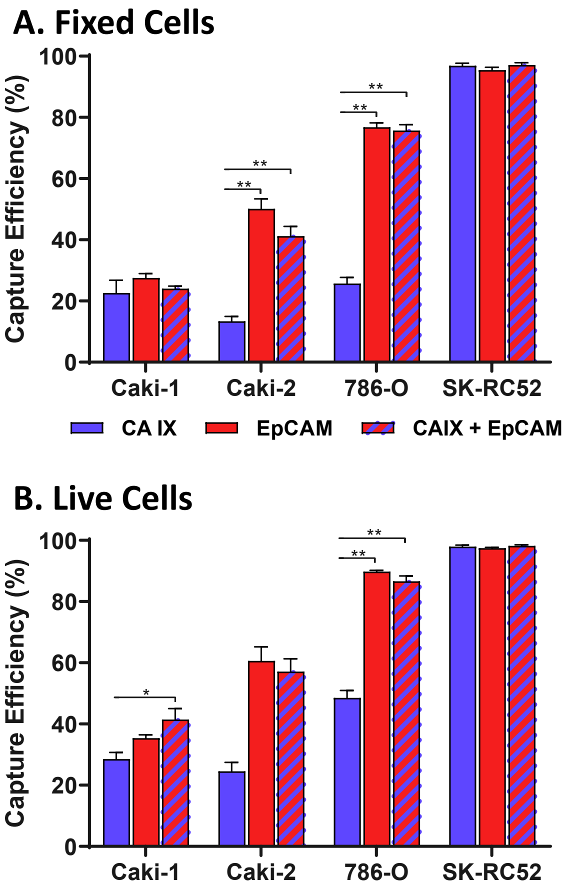

Supplemental Figure 2: The percentage of captured cells was evaluated on four RCC cell lines after fixation (A) and live (B) using indirect SeraMag binding with antibodies to CAIX (blue), EpCAM (red), or CAIX and EpCAM (red/blue). Significant differences in capture efficiency within each cell line were detected using a one-way ANOVA with multiple comparison ($p<0.05$(*), $p<0.01$ (**)).

Methodology	Antibodies Used	Manufacturer
Flow Cytometry	CAIX	R&D Systems
	EpCAM	R&D Systems
	CAXII	Cedarlane Labs
	pCK	Biolegend
CTC Capture	CAIX	R&D Systems
	EpCAM	R&D Systems
Fluorescence Microscopy on CTCs	CAXII	Cedarlane Labs
	pCK	Biolegend

Supplemental Table 3: Overview of which antibodies were used for the different methodologies.

Patient ID	CK S+	Double +	CAXII S+
1	32.3%	62.2%	5.4%
2	79.5%	14.1%	6.4%
3	36.8%	45.6%	17.5%
4	70.3%	18.8%	10.9%
5	65.9%	9.8%	24.3%
6	47.5%	42.3%	10.2%
7	92.1%	2.1%	5.9%
8	86.3%	2.4%	11.3%
9	75.0%	0.0%	25.0%
10	8.8%	25.0%	66.3%
11	0.0%	0.0%	100.0%
12	0.0%	6.1%	93.9%
13	1.6%	37.9%	60.5%
14	56.5%	26.1%	17.3%
15	18.5%	32.5%	49.0%
16	12.5%	29.4%	58.1%
17	72.3%	19.9%	7.8%
18	50.0%	13.0%	37.0%
19	0.0%	60.6%	39.4%
20	50.0%	0.0%	50.0%

Supplemental Table 4: Composition of CTC sub-populations for the 20 patient cohort. CTCs were separated into three groups: CK Single+, Double+, and CAXII Single+.

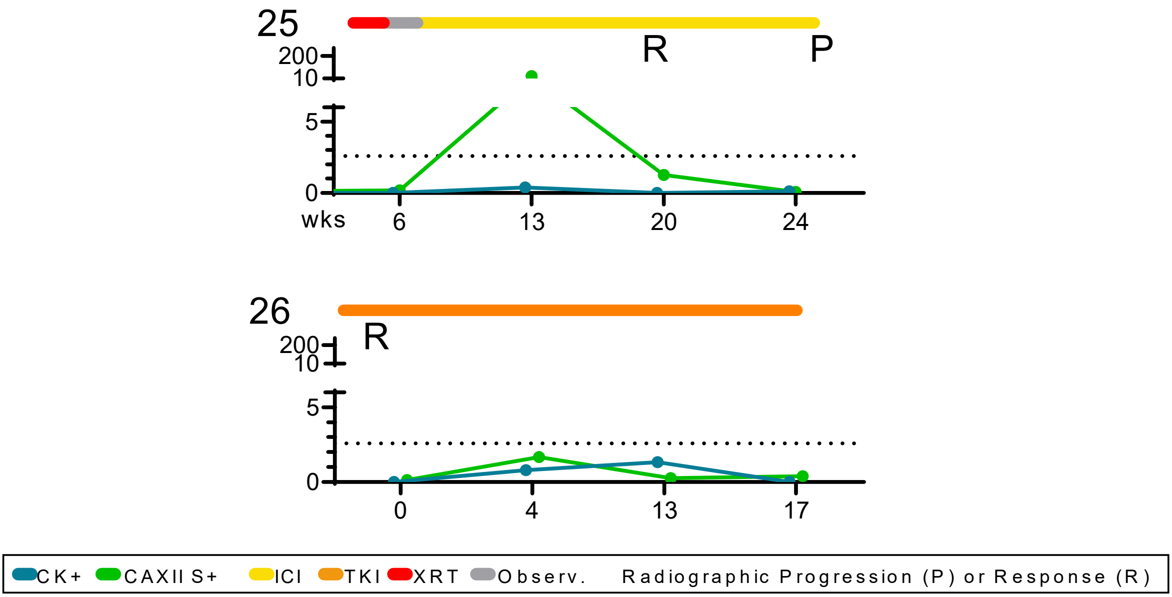

Supplemental Figure 3: CTC biomarker evaluation of two additional patients (#25 and 26) over time compared to therapeutic history (colored bars) for each patient. Graphs represent enumeration for each CTC population. Dashed line represents the optimal cutoff for CK+ CTC number (2.6) identified in figure 2. Patient 25 received radiation during the initial CTC evaluation, followed by a spike in the number of CAXII single+ CTCs after beginning combination Ipi/Nivo. This population of CTCs declined during subsequent cycles of treatment, perhaps reflecting an initial flare of cells in circulation. Multiple new brain metastases developed at the final blood draw, while systemic response to treatment was maintained in liver, lung and lymph node metastases suggesting that disease progression in a sanctuary site such as the central nervous system may not be reflected in tumor cells in circulation. Patient 26 was treated with pazopanib for more than 9 months prior to the first CTC evaluation. Less than 3 CTCs were detectable at any time point during treatment and after 17 weeks of tracking, the patient discontinued pazopanib due to other medical issues.
